# Coughing-induced bowel transection in a patient with an incarcerated inguinal hernia: a case report

**DOI:** 10.1186/1752-1947-7-47

**Published:** 2013-02-15

**Authors:** Hekmat Hakiman, Jana DeLibero, Thai Pham, Sean Dineen, Sergio Huerta

**Affiliations:** 1 University of Texas Southwestern Medical Center Department of surgery, 5323 Harry Hines Blvd., Dallas, TX, 75390-9159, USA; 2 VA North Texas Health Care System 4500 S., Lancaster Road, Dallas, TX, 75216, USA

**Keywords:** Bowel transection, Hemoperitoneum, Inguinal hernia

## Abstract

**Introduction:**

Although blunt trauma to a hernia-containing bowel is known to cause bowel perforation, this report documents the first incident of a small bowel transection following a non-traumatic event.

**Case presentation:**

We report the case of a 49-year-old African American man with a chronic incarcerated inguinal hernia awaiting elective repair. He presented to the Emergency Department with abdominal pain following an episode of coughing. On examination, he was found to have peritonitis. He underwent exploratory laparotomy, and had a complete small bowel transection. A bowel resection with primary anastomosis was performed, as well an inguinal hernia repair.

**Conclusion:**

Chronic hernia incarceration can lead to weakening and ischemia of the bowel, and minimal trauma can lead to perforation of the weakened segment. In such presentations, bowel resection and repair of the defect with a biological material is safe and feasible.

## Introduction

The lifetime risk for development of inguinal hernias is 27% and 3% for men and women, respectively [1-3]. Although some studies have demonstrated that small, asymptomatic inguinal hernias might be observed [4,5], the mere presence of an inguinal hernia is an indication for surgical repair by most surgeons. Thus, most hernias are electively repaired provided that the operative risk is not prohibitive. Complications from inguinal hernias include pain, strangulation, and bowel obstruction. The most serious complication of an inguinal hernia is strangulation as this might lead to bowel ischemia and perforation.

A prospective study inclusive of 669 subjects demonstrated a 0.3% rate of bowel or omentum resections at elective repair of inguinal hernias [6]. Data documenting the rate of incarceration is available only from small cohort and retrospective studies. The rate of strangulation, however, is less than 1% per year from collective data in all patients presenting with an inguinal hernia [6-10]. This rate, however, is substantially higher in patients who have a history of an incarcerated hernia (4% to 5%).

In the present report, we describe a patient with a large inguinal hernia who developed bowel transection following a coughing episode. The rarity of this event is discussed along with the elective repair during the same setting.

## Case presentation

A 49-year-old African American man presented to a clinic with an 18-month history of a painful, enlarging right groin bulge. On physical examination, he had a large right inguinal hernia containing chronically incarcerated bowel with a scrotal component. He denied any history consistent with obstructive symptoms. However, he expressed interest in surgical intervention as the hernia continued to increase in size and started causing symptoms.

While awaiting elective surgical repair, he presented to the Emergency Department (ED) with acute onset of right groin and abdominal pain. Upon arrival to the ED, he indicated that the symptoms began after he had gone to the bathroom for a bowel movement. Following his attempted continuation of bed rest, he began an aggressive bout of coughing. There was no history of trauma to his groin or abdomen. Following the coughing episode, he developed sharp, intense right groin pain and an episode of non-bloody, non-bilious vomiting.

In the ED, his physical examination demonstrated hypertension (182/124 mmHg) and normocardia (99 beats per minute). Further examination demonstrated that his abdomen was distended and rigid as well as diffusely tender to mild palpation.

Laboratory evaluation showed hypokalemia (3.6 mmol/L), but no other abnormalities. His white blood cell count was 8000 K/cu mm and his lactic acid was 1.3 mEq/L. An upright chest radiograph showed no free air.

**Figure 1:**
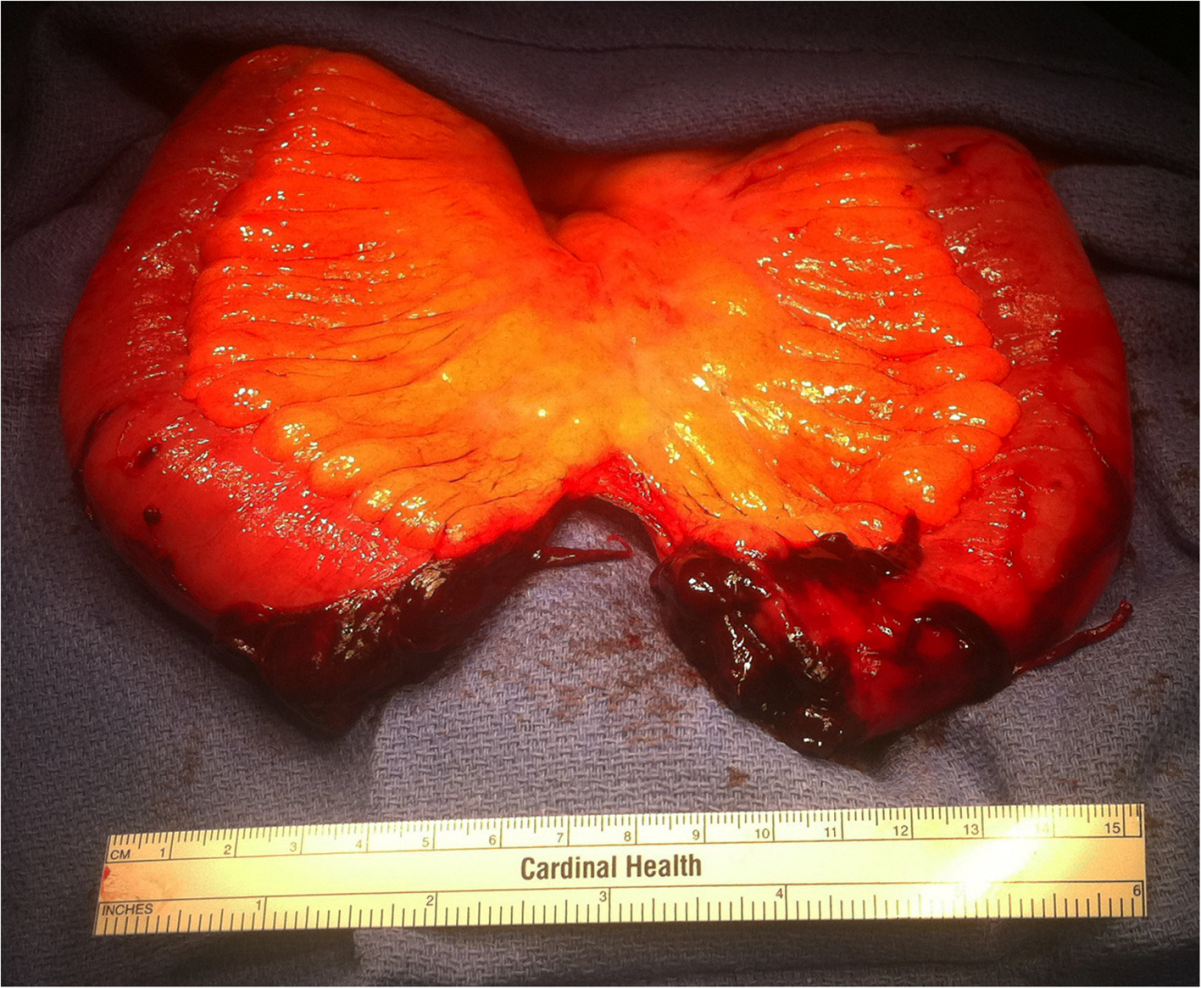
Gross specimen demonstrates transected jejunum approximately 50cm from the terminal ileum.

Given that his abdominal examination was consistent with peritonitis, he was taken to the operating room for an exploratory laparotomy. At celiotomy, there was a large amount of blood (approximately 400 cc) mixed with a small amount of feculent material. A segment of small bowel was found within the inguinal canal. Following lysis of adhesions and extraction of the bowel from the inguinal canal, a complete circumferential segment of jejunum was identified (Figure 1). No other bowel injuries were noted.

A small bowel resection was performed on both sides of the transection with a gastrointestinal anastomosis device followed by a side-to-side two layer hand-sewn anastomosis.

The patient had excellent hemodynamics and attention was directed at the inguinal canal through a separate groin incision. The inguinal canal demonstrated an obliterated transversalis fascia. The hernia sac was addressed via the transabdominal portion of the operation. We then proceeded with repair of the inguinal floor from the anterior incision. This was undertaken with biologic mesh (AlloDerm®) using the Lichtenstein technique. The remainder of his hospital course prior to discharge was uneventful. There was no surgical site infection at discharge or during clinic follow up. No recurrence had been noted up to 18 months following repair.

## Discussion

Inguinal hernia repairs comprise a large portion of general surgical procedures. There are more than 20 million hernias estimated to be repaired annually around the world [2,8]. In the USA, an inguinal herniorrhaphy is the most common elective operation performed with approximately 700,000 cases annually [2,8].

The vast majority of hernias are repaired electively to prevent complications. Although complications are rare, they might be severe if not promptly addressed. Incarceration, strangulation and bowel perforation are known complications of inguinal hernias. Expectedly, intestinal perforation is higher in patients with inguinal hernias compared with the general population [11]. This is typically the result of an incarcerated hernia leading to strangulation. Small bowel tears and perforations have been reported in the setting of an inguinal hernia, usually following direct trauma to the patient’s abdomen or groin [12,13]. To the best of our knowledge, small bowel perforation or transection has not been reported following a non-traumatic insult to the hernia such as coughing.

Non-traumatic events leading to intestinal transection are rare events. Experimental models demonstrated that blunt trauma to an inguinal hernia could produce enough force (300mmHg) to lead to intestinal pressures of 260mmHg that can cause intestinal disruption [14]. By contrast, urodynamic testing comparing voluntary cough to laryngeal cough reflex revealed that the maximal intraluminal pressure generated was only 110mmHg [15]. This pressure is not generally sufficient to cause intestinal disruption.

In the present case, it is possible that the chronic incarceration of the hernia might have led to bowel edema, weakening of the bowel wall and the observed transection. There were no other identifiable risk factors in this patient that could have led to this outcome.

In the present report, we elected to proceed with the repair of the inguinal hernia during the same operation. Although the timing of hernia repair in regards to the index operation is controversial, the status of the patient is a universal issue of concern in determining whether to proceed with a repair or not. However, if hemodynamic stability is well established, some authors favor a delayed repair [12,16] and others prefer a repair in the same setting [17,18]. Because the patient in this report had physical examination findings consistent with peritonitis, there was a clear need for operative intervention and the hernia was repaired because the patient was hemodynamically stable. In addition, the patient did not have any comorbid conditions that could lead to cardiopulmonary morbidity related to increased operative times. A balance between two operations and increased morbidity in a lengthy operation must be carefully considered on a case-to-case basis.

This operation would be classified as a contaminated case based on wound infection classification. Thus, the use of synthetic mesh was considered a poor option as this leads to high rates of infection, bleeding and fistula formation [19]. A tissue repair has been advocated in this setting [20]. However, tissue repair is associated with a high rate of recurrence. Because the transversalis fascia in this patient was obliterated, a tissue repair was not optimal. We thus elected to repair the floor with biologic mesh (AlloDerm®), which has been described in similar settings [21].

## Conclusion

The present report describes an unusual complication of an incarcerated hernia in a young man. It is unclear what led to the weakening of the bowel wall causing a transection with a small increase in intraluminal and intra-abdominal pressure such as that produced by coughing. Repair of the floor with a biologic mesh in the same setting was feasible in this case and has produced good results to date.

## Consent

Informed consent was obtained from the patient for publication of this manuscript and accompanying image. A copy of the written consent is available for review by the Editor-in-Chief if this journal.

## Competing interest

The authors declare that they have no competing interest.

## Authors’ contribution

HH, JD and SH were involved in the initial writing of the manuscript. SH provided major editing changes. HH, JD, TP were primarily involved in the care of the patient. TP and SD provided intellectual contributions to the content of the manuscript as well as editorial assistance. All authors have read and approved the final version of the manuscript.
